# Fading distributed scaffolds: the importance of complementarity between teacher and material scaffolds

**DOI:** 10.1007/s11251-018-9474-0

**Published:** 2018-11-07

**Authors:** Nicole D. Martin, Catherine Dornfeld Tissenbaum, Dana Gnesdilow, Sadhana Puntambekar

**Affiliations:** Department of Educational Psychology, University of Wisconsin - Madison, 1025 W Johnson Street, Madison, 53706 WI USA

**Keywords:** Scaffolding, Distributed scaffolding, Fading, Teacher mediation

## Abstract

Designing learning environments with *distributed scaffolding*—support distributed across different instructional tools, activities, and the teacher—can help support students’ different needs, but a critical question is how the design incorporates the hallmark feature of responsive support. While most material scaffolds in instructional tools are inherently static, teachers can complement support provided in material scaffolds by providing responsive assistance and mediating students’ interactions within their environment to both support and challenge students. Our study explores the interplay between support embedded in instructional materials and scaffolding provided by teachers. We focused on how teachers’ scaffolding complemented the fading material scaffolds in a paper-and-pencil tool and how this combination of support impacted students’ learning of science practices and content. Differences in teachers’ responsive versus static scaffolding moves corresponded with differences in students’ performance as material scaffolds faded in support. One teacher complemented support provided by the material scaffolds by frequently monitoring students’ understanding and providing additional support as needed, even when material scaffolds faded; her students maintained a high level of performance throughout the unit. In contrast, the other teacher tended to extend the static kind of scaffolding found in the instructional materials rather than adapt support to his students’ needs as material scaffolds faded; his students showed a significant decrease in performance over time. Our findings show that the complementarity between responsive scaffolding moves from the teacher and scaffolding embedded in instructional materials is important for effectively supporting the wide range of students’ needs in the classroom.

In classrooms where students work on complex problems or projects (Hmelo-Silver and Barrows 2006; Kolodner et al. 2003; Reiser et al. 2001), support or *scaffolding* is often provided through instructional materials or technology. Scaffolding describes the pedagogical support that is calibrated to a learner’s current level of understanding and helps the learner accomplish tasks that he or she could not accomplish alone (Wood et al. 1976). This difference between what a learner can accomplish alone and with assistance is known as the zone of proximal development, or ZPD (Vygotsky 1980). The ZPD describes a range of understanding for a student in which targeted support can balance sufficient challenge for students while preventing boredom and frustration. The original notion of scaffolding assumed that a single more knowledgeable person, such as a parent or teacher, would support an individual learner, providing exactly the help he or she needed to move forward (Bruner 1985). But very often in classroom contexts, support is the same for all students and is not tailored to each student’s needs for particular tasks in which they are engaged. Support provided through tools, or *material scaffolds*, including instructional materials and technology, fulfills a key function in that all students can move forward on their goals. But support provided by peers and teachers, or *social scaffolds*, play a crucial role in extending and complementing the support that is provided in tools so that each student’s needs are met (Tabak 2004; Tabak and Reiser 1997; Puntambekar et al. 2007). The idea of distributed scaffolding, in which various types of tools, routines, and activities are used to support a range of students, is now being increasingly applied in classroom contexts (Puntambekar and Kolodner 2005; McNeill et al. 2006; Tabak 2004; Luckin 2010). Material scaffolds including technology tools (Linn et al. 2003; Luckin and Du Boulay 1999) and written prompts in paper-and-pencil tools (McNeill et al. 2006) are being used alongside social scaffolds, such as support provided by teachers or peer interactions (Kolodner et al. 2003; Palincsar and Brown 1984; Palincsar et al. 1993). However, one of the key challenges of implementing distributed scaffolding is that the agents, tools, and resources must complement one another, so that they all work together in a system of scaffolding (Pea 2004; Smagorinsky et al. 2015; Tabak 2004).

Paper-and-pencil tools are increasingly being used as material scaffolds because they can be easily integrated in classroom teaching, are inexpensive, and therefore can be widely used in schools with limited access to resources, such as few computers for use by hundreds of students. But paper-and-pencil tools often provide a fixed set of prompts that are the same for all students in a classroom, and thus, may need to be complemented by a source of more dynamic support. A key feature of scaffolding is that it is temporary and eventually fades as learners take more responsibility for their learning (Pea 2004). Additionally, the level of support is tailored or calibrated to the student’s knowledge and understanding at any particular point in time. Both fading and calibrated support are hard to achieve in paper-and-pencil tools. Even if some level of fading is built into these tools, it is the same for all students, and not sensitive to the level of understanding of individual learners. Teachers, therefore, play a crucial role in dynamically extending and complementing the support embedded in instructional materials, changing both the amount and quality of support based on their students’ needs (Belland et al. 2015; Ge and Land 2004; Puntambekar et al. 2007; Saye and Brush 2002; Tabak 2004).

Our focus in this paper has been on understanding how a paper-and-pencil tool, a material scaffold, which we call the Scientist’s Journal, is used by the teacher. Our aims in this study were two-fold. First, we wanted to understand how we could build gradual fading into a paper-and-pencil tool such as the Scientist’s Journal, which, by nature of its being printed, provided support that was static. Based on our prior work and understanding of students’ difficulties in doing science, we wanted to understand how we could gradually fade the support provided by the Scientist’s Journal. Our second aim was to understand how teachers used and adapted the Scientist’s Journal in their teaching, and ways in which their ongoing support complemented the support provided in the Scientist’s Journal.

This paper is organized as follows: first, we discuss material scaffolds and social scaffolds, specifically from teachers, as well as the interplay between these types of scaffolds. We then describe the research questions, methods, and findings of our study. We conclude by discussing the implications of this work for understanding complementarity between material and social scaffolds to inform the improved design of instructional materials and professional development strategies for teachers.

## Material and social scaffolds

The original description of scaffolding by Wood et al. (1976) foregrounded the dynamic and responsive characteristics of scaffolding provided by a more knowledgeable other, such as a teacher. In this description, the tutor used a variety of techniques, including prompting, questioning, and modeling. However, in classrooms where students engage in solving ill-structured problems, instead of a single tutor providing many forms of support, multiple tools and practices are now used to scaffold learning. Each tool may be designed to support a specific task or multiple tools may support a single task (Pea 2004; Puntambekar and Kolodner 2005; Tabak 2004). Saye and Brush (2002) conceptualized this scaffolding in two forms: *hard* and *soft* scaffolds. *Hard scaffolds* described “static supports that can be anticipated and planned in advance,” such as embedded prompts, while *soft scaffolds* described spontaneous scaffolding from the teacher (Saye and Brush 2002, p. 81). Similarly, Pea (2004) described *social* scaffolding as support provided by another person contingent on the learner’s needs and *technological* scaffolding as designed artifacts that support learning. In classroom situations, social scaffolding can be provided by a teacher or peers.

Our focus in this paper is on scaffolding provided by teachers as they supported the students’ use of the Scientist’s Journals. We further elaborate on the nature of material scaffolds and teacher scaffolding in the next sections. We also highlight the importance of complementary interactions between material and teacher scaffolds, or interplay, in the process of supporting students.

## Material scaffolds

We define material scaffolds as support purposefully designed and embedded within instructional materials, such as printed activities and technology tools, to help students work through complex problems. Material scaffolds often address anticipated difficulties that students might have (Quintana et al. 2004) so that students may accomplish what would otherwise be beyond their ZPDs (Luckin 1998, 2010; Reiser 2004). One mechanism by which material scaffolds support learning is *structuring*. Structuring helps students by decomposing a complex task into the necessary steps to complete it (Reiser 2004), such as by providing guidance for participating in authentic scientific practices (Belland et al. 2015; Xenofontos et al. 2018). Structuring includes providing organizational mechanisms to help students complete a task or engage in practice, including questions that support reflection and articulation.

Material scaffolds have also been designed to provide *prompts* to help students complete a task. Researchers have examined the effect of various types of prompts to support learning, reasoning, and reflection (e.g., Ge and Land 2004). Paper-and-pencil tools have been designed with specific prompts to help students focus on the conceptual underpinnings of a task (Puntambekar and Kolodner 2005). Similarly, computer-based scaffolds such as the WISE environments (Linn et al. 2003) provide prompts that guide students through the inquiry process. In addition to fixed or static prompts, prompts that adapt to the learner’s current needs have also been used, although the adaptation is not at the same level as a human tutor would provide. For example, Ecolab (Luckin and Du Boulay 1999), based on the notion of the ZPD, provides layers of scaffolding in an interactive environment. Additionally, intelligent tutoring systems and cognitive tutors may adapt to students’ understanding and provide less support over time as needed (Azevedo et al. 2016; Lajoie 2005; Koedinger and Corbett 2006; Taub et al. 2014). However, such layers of scaffolding and adaptations are still pre-planned and based on expected difficulties and misconceptions students may have. This support can be useful, but may not address the unique needs of individual students. If a student’s understanding does not align with the expectations of the technology, then the system cannot ask the nuanced follow-up questions that a human can to figure out how to help the student.

In classrooms, material scaffolds can play an important role in providing support for many students with different needs. Since the goal of providing support to students is to effectively prepare students to eventually complete tasks without support, it is important to consider how fading may be built into instructional materials. Fading of support ensures that students can act independently and articulate knowledge of their own accord (Lajoie 2005). From a distributed cognition perspective, fading can be accomplished by removing individual material scaffolds over time, once their key purpose has been served (Belland 2011). As an example, McNeill and colleagues (McNeill and Krajcik 2009; McNeill et al. 2006) designed scaffolds in printed instructional materials to help students write scientific explanations. They examined the effect of fading the material scaffolds and found benefits of faded support over constant support. Also, Ge and Land (2004) incorporated question prompts that transitioned from procedural prompts with explicit task-based support to reflective prompts with meta-level support, which helped students understand how, when, and why to engage in cognitive and metacognitive processes.

Knowing how and when to integrate fading into instructional materials is a significant challenge (Lajoie 2005), especially for printed materials. In our study, we integrated fading into the Scientist’s Journals by including three levels of support distributed over multiple experiments that students conducted to solve a design challenge. Experiments in the beginning of the unit provided high support, in which hypothesis writing and data interpretation activities were highly structured in that prompts included predefined responses for students to choose from. Experiments in the middle and end of the unit provided medium and low support, respectively, as these activities and prompts became more open-ended, giving students more responsibility to generate their own ideas.

When incorporating fading into the Scientist’s Journal, we kept in mind that these material scaffolds were only one part of a system of support within the classroom (Puntambekar and Kolodner 2005; Tabak 2004). Students may access other modes of support in the event that the built-in fading progresses too quickly for them. When the predetermined level of support in material scaffolds is insufficient, teachers are uniquely positioned to manage this apparent lack of support (Belland et al. 2015; Ge and Land 2004; Saye and Brush 2002). Even students with access to adaptive technology tools that adjust the level of support to different students’ needs benefit from the fine-grained calibration and sensitive tailoring of support that a human teacher can provide (Azevedo et al. 2011; Koedinger and Corbett 2006). To understand how teachers successfully provide support that complements material scaffolds, we must understand the synergy between the scaffolding provided by teachers and the predetermined support in materials scaffolds.

## Teacher scaffolding

Teacher scaffolding offers relatively personalized support in response to students’ ZPDs as they work in a classroom. While material scaffolds are often predefined and identical for all students (Puntambekar and Hübscher 2005), teacher scaffolding can adapt in the moment and directly respond to students’ understanding. For example, Ge and Land (2004) found that the teacher could effectively guide students’ use of material scaffolds in the classroom, although providing this guidance is challenging for teachers in classrooms with many students (Belland et al. 2015).

Teacher scaffolding involves continual diagnosis of students’ understanding, responsive support, and eventual handover of independence to students (Smit et al. 2012). To do this, teachers can ask questions to elicit students’ ideas and to assess understanding (Chin 2007). Based on this diagnostic process, teachers can make decisions about providing more or less support that is tailored to students’ understanding (van de Pol et al. 2014). For example, van de Pol et al. (2014) successfully developed a model of contingent teaching that helped teachers to improve their diagnostic strategies and make conscious, careful decisions about when to increase or fade support, depending on students’ responses to prompts. Contingency was also highlighted as an essential characteristic of scaffolding in van de Pol et al. (2010) review of scaffolding research. Ge and Land (2004) found contingency in teachers’ scaffolding when teachers adjusted their support as material scaffolds transitioned from procedural to reflective questions. Teachers might also fade their support during class discussions by minimizing their role as students take greater responsibility for continuing the discussion (Forman et al. 2017; Tabak and Baumgartner 2004). Responsive, personalized scaffolding within each student’s ZPD is effective for learning and is often recognized as a mark of excellent teaching (van de Pol et al. 2010, 2014).

While the original notion of scaffolding described one-on-one support, recent research has helped conceptualize critical features of scaffolding for whole classrooms, in which a teacher engages in ongoing diagnosis of understanding, responsive strategies, and handover of independence to a collective group of students (Smit et al. 2012). Smit et al. (2012) investigated the extension of the scaffolding metaphor in whole classrooms by studying these key characteristics of scaffolding over nine language lessons in a primary school. The teacher enacted these characteristics both during lessons and between lessons, described as layers of support (Smit et al. 2012). Smit et al. (2012) also noted that, in addition to this layering of support, the teacher’s support was distributed over time, which, in combination, had a cumulative effect.

In sum, monitoring multiple ZPDs and simultaneously providing calibrated support to different students in a classroom is challenging for teachers (Brown et al. 1993; Kolodner et al. 2003). To address this challenge, teachers may integrate material scaffolds, such as instructional materials or technology, into their teaching to provide support to a larger group of students (Puntambekar and Kolodner 2005). But there are different implications for different combinations of scaffolds and the interplay between them (Ge and Land 2004; Tabak 2004; Belland et al. 2015). We therefore need to understand better how teachers use material scaffolds in their classes, both to help us design better scaffolds, as well as to provide practical suggestions to teachers.

## Interplay between distributed scaffolds

As described earlier, distributed scaffolding involves carefully integrating support across multiple instructional tools, activities, and agents, including the teacher (Puntambekar and Kolodner 1998, 2005), to create a system that simultaneously supports all students in a classroom. Distributed scaffolding may include redundancy and/or synergy creating multiple opportunities for a range of students’ needs (Reiser and Tabak 2014; Tabak 2004). To successfully design distributed scaffolding and support students in a classroom, we need to better understand the functions of each type of scaffold, and how scaffolds can work together. There might be different combinations of scaffolds that work in different contexts, for different tasks, or for the same task at different points in time. The particular combination of scaffolds that our study examined is that between materials scaffolds with static levels of fading and the scaffolding provided by teachers.

A critical question involved in the design of scaffolding is how best to incorporate the hallmark feature of responsive and adaptive support. While paper-and-pencil tools and software may include embedded scaffolds for anticipated areas of difficulty, the “blanket” support provided by material scaffolds may not align with individual students’ ZPDs, as the support is identical for all students (Puntambekar and Hübscher 2005). Teachers, however, can modify the use of material scaffolds by providing responsive instruction that gives more or less help when needed, even when material scaffolds remain static and unchanging (Ge and Land 2004). Thus, teachers play a vital role in mediating the use of tools and instructional materials during the dynamic process of scaffolding. Teachers are involved in a participatory relationship with the material scaffolds in their classrooms and can orchestrate how these scaffolds are utilized (Ge and Land 2004; Remillard 2005). Verbal scaffolding from teachers can more dynamically cater to students’ needs than static, predefined scaffolds and can complement these material scaffolds to support students’ learning (Saye and Brush 2002; Songer et al. 2012).

Research suggests that the scaffolding provided by teachers is crucial in situations where some of the support is embedded by technology tools. Azevedo et al. (2005) have developed what they refer to as fixed and adaptive scaffolds in hypermedia environments to promote students’ self-regulated learning. *Fixed scaffolds* involve fixed interface structures in the form of domain-specific questions or sub-goals, whereas *adaptive scaffolds* consist of guidance provided by a human tutor. They found that students who received adaptive scaffolds outperformed students who received fixed scaffolds. The beneficial interplay between teacher and technology has also been described by Kim and Hannafin (2011) and Raes et al. (2012) for the WISE environment. Kim and Hannafin (2011) described different kinds of scaffolding provided by teachers, peers, and technology, while Raes et al. (2012) described how the inclusion of both teacher- and technology-enhanced scaffolding was essential for students with low prior knowledge. Even learning environments that utilize adaptive technologies to provide material-based support are more effective when teachers add complementary support, especially for students with low prior knowledge. Studies by Schofield et al. (1994), Koedinger and Corbett (2006), and Epstein and Hillegeist (1990) demonstrated that teachers and pedagogical agents (e.g., cognitive tutors, intelligent tutoring systems) have complementary roles in the classroom. Incorporating a pedagogical agent off-loaded certain components of instruction, allowing teachers to provide more individualized support to students. More recent studies have indicated that adaptive technologies still benefit from a human tutor. Adding a human tutor to an adaptive hypermedia environment resulted in greater knowledge gains than the environment on its own (Azevedo et al. 2011), and pedagogical agents designed to mimic human tutors failed to completely meet the needs of students with lower prior knowledge (Azevedo et al. 2016).

While material scaffolds can address the anticipated difficulties that students might have (Quintana et al. 2004) and some can provide adaptive support through technology, they may not meet a student’s individual needs in the moment (Belland et al. 2015). Responsive support from teachers that changes dynamically can extend the support provided in material scaffolds (Lajoie 2005; Puntambekar and Hübscher 2005). Teachers can provide responsive assistance and mediate students’ interactions within their learning environment to ensure that students are both supported and challenged (Kozulin and Presseisen 1995; Tabak and Reiser 1997). The interplay between teacher and material scaffolding can vary in its balance and timing, especially in how the teacher builds connections between materials and activities (Puntambekar et al. 2007). For example, teachers might interleave whole-class discussions before or after students’ work with the material scaffolds (Tabak and Reiser 1997). Teachers may also engage with small groups of students as they work with material scaffolds. Teacher mediation may entail monitoring the pace and effectiveness of static scaffolds and responding with support when static scaffolds are not completely aligned with learners’ ZPDs (Puntambekar et al. 2007). From the distributed scaffolding perspective, the coordination and mediation of material scaffolds is essential for successfully supporting students with differing ZPDs (Belland 2011; Belland et al. 2015). However, the interplay between teacher and material scaffolds to mediate students’ learning is still not well understood (Lajoie et al. 2001).

## The present study

Our study addresses the issue of how teachers make use of material scaffolds to support their students by investigating the interplay between support embedded in instructional materials and scaffolding provided by teachers, and how this interplay affects students’ learning outcomes. In particular, we focused on how scaffolding from two teachers complemented the fading material scaffolds in the Scientist’s Journal and how this combination of support impacted students’ learning of science practices and content. This study aimed to answer the following research questions:

- How do material scaffolds impact students’ learning when support is gradually faded in a predetermined and uniform way for all students?
- What role does teacher scaffolding play in classrooms when support is also provided by material scaffolds?

To answer these questions, we evaluated how two teachers working with the same curriculum adjusted their scaffolding for students as material scaffolds faded in support.

## Methods

We investigated scaffolding within a design-based curriculum that promotes science learning through distributed scaffolding (Puntambekar et al. 2007). The curriculum spanned 10 weeks and presented students with the central challenge of designing a fun, safe, roller coaster ride. To solve their challenge, students needed to learn about physics concepts related to forces, energy, work. Students used a Scientist’s Journal, an online digital text, a roller coaster simulation, and hands-on experiments to investigate these concepts. All the activities within the curriculum were connected and framed to help students solve the roller coaster design challenge. Details about the aspects and sequence of the unit are shown in Table 10 (Appendix). The Scientist’s Journal, described in the data sources section, provided prompts for students to engage in the practices of science, such as hypotheses writing, data recording and making claims based on data. Classroom discussions led by the teacher were video-recorded throughout the unit.

This study was part of a larger implementation that especially focused on schools in inner city and resource poor areas. The implementations themselves were at the phase of expanding to rural and resource-poor areas and testing our innovation in different, and perhaps more challenging, contexts as part of a Design-Based Research paradigm (Design-Based Research Collective 2003). We examined the specific questions of this study within the larger implementations.

## Participants

Two teachers, Mrs. Lewis and Mr. Green (pseudonyms), and their sixth-grade students participated in the study (*n* = 28 and *n* = 44, respectively). This was the fourth year that both teachers had worked with the research team using similar design-based curricula. Both teachers had previously participated in several professional development workshops designed to help them support students’ open-ended inquiry. Both teachers taught in a suburban, public middle school of a mid-sized city in Midwestern US. Approximately 85% of students at this school were Caucasian, and 19% were eligible for free or reduced-price lunch. The participating science classes were composed of students with mixed abilities and met daily for 45 min during the unit.

### Data sources

We used three data sources: (a) students’ work in their Scientist’s Journals; (b) students’ scores on physics and science practices pre- and post-tests; and (c) videos of classroom discussions to examine teachers’ scaffolding.

#### Scientist’s journals

We used available student data from all consenting students in Mrs. Lewis and Mr. Green’s three science classes who completed all parts of the Scientist’s Journals analyzed in this study (i.e., no absences or blank responses during the unit). The Scientist’s Journals served as material scaffolds and included support that was gradually faded over the course of the unit. This fading of support was embedded in two activities that focused on the science practices of hypothesis writing and data collection. These were repeated for nine experiments during the unit for a total of nine hypothesis-writing and data-collection activities. At the end of each experiment, students reported whether their results supported their hypotheses or not, and provided explanations using data and evidence from their experiments, referred to as “Report Out” activities.

Of the nine experiments, the first three included *High Material Support*, the middle three included *Medium Material Support*, and the last three included *Low Material Support*. Table 1 shows a timeline of this fading. In the High Material Support experiments, hypothesis writing was highly scaffolded so that students circled the predicted outcome from three options. Students were also given partially completed data charts that included the necessary independent and dependent variables. In the Medium Material Support experiments, hypothesis writing was re-structured so that students completed a prompt by filling in a blank, which allowed for more open-ended responses. This section also contained partially completed data charts that included independent and dependent variables, along with blank data charts that required students to identify and organize variables within the chart. In the Low Material Support experiments, the hypothesis activities were open-ended and gave students the opportunity to write their own hypotheses as full sentences. Further, all data charts in these experiments were blank, requiring students to identify variables and organize their charts for data collection. This fading was based on our prior work and understanding of difficulties students face in science. Table 1 describes the goals of each scaffolding level and provides examples.

**Table 1:**
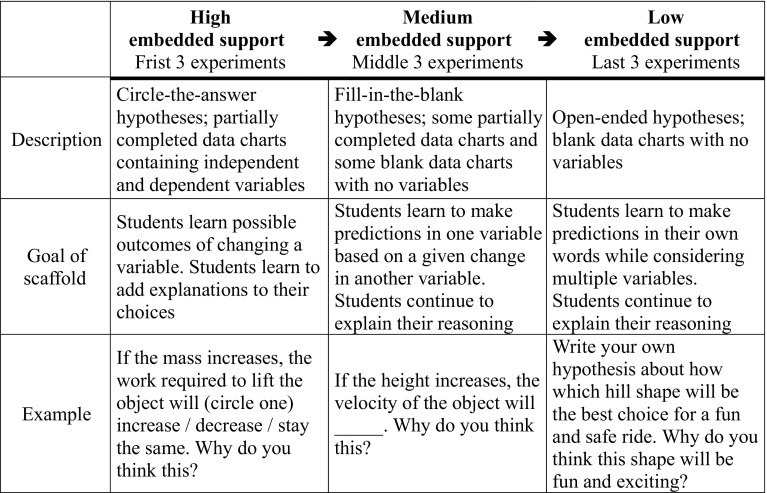
Description of material scaffolding levels

#### Content and practices tests

The physics conceptual test assessed students’ knowledge about physics concepts related to forces and motion, while the science practices test assessed students’ ability to engage in scientific reasoning and data interpretation. The physics conceptual test consisted of 29 multiple choice questions that assessed students’ understanding of physics concepts related to forces, motion, and energy, with a maximum score of 29 points. Internal validity checks on the physics test resulted in a Cronbach’s Alpha = .76, indicating acceptable internal consistency.

The science practices test consisted of 14 multiple-choice and 4 open-ended questions that assessed students’ ability to engage in scientific reasoning and data interpretation, with a maximum score of 22 points. The open-ended questions were coded on a 0–2 point scale depending on the quality of students’ responses. Internal validity checks on the science practices test resulted in a Cronbach’s Alpha = .89, indicating good internal consistency. The pre- and post-tests were identical.

#### Classroom videos

We selected one target class for video recording for each teacher based on the teachers’ feedback about which class was most academically representative. We selected 50 video recordings of whole-class discussions over the entire curriculum (26 from Mrs. Lewis’s classroom (8 h, 29 min) and 24 from Mr. Green’s classroom (8 h, 14 min). We chose these videos to examine how teachers scaffolded students’ learning during whole-class discussions. We selected whole-class discussions that occurred before and after each simulation experiment, when the teacher and students prepared for the experiment and reviewed their results together. The teachers also used whole-class discussions to elaborate on specific concepts and relationships.

### Analysis

#### Scientist’s journals

We focused our analysis of students’ performance on the culminating Report Out activities, which included both hypothesis writing and data interpretation. The Report Out activities were designed to help students learn to report results using data as evidence for claims. Two researchers coded students’ Report Outs (*n* = 28 for Mrs. Lewis, *n* = 44 for Mr. Green) for (a) the correctness of connections to previous hypotheses, (b) explanation of key science ideas, and (c) use of data to support hypotheses. The coding rubric is shown in Table 2; each Report Out section received a score between 0 and 3. The researchers achieved “almost perfect agreement” with a Cohen’s kappa of .82 on 20% of the data (Stemler 2001). Disagreements were resolved through discussion, and the remaining data was coded by one of the researchers. We analyzed the Report Out scores with an ANCOVA using a Holm-Bonferroni correction to account for family-wise error in multiple comparisons. The teacher was the independent factor, and covariates included pre-test scores on the physics conceptual and science practices tests. Research suggests that differential prior knowledge impacts how students benefit from scaffolds (Belland et al. 2015). In the case of the Report Out activities, students needed to identify variables and explain their data based on physics content knowledge and competency in scientific practices. Thus, we used students’ pre-intervention scores on the physics conceptual test and science practices test as covariates in our analysis to account for differences in prior knowledge and ability.

**Table 2:** Coding rubric for “Report Out” activities

Score	Description of score
0	No response Mismatch between initial hypothesis and explained hypothesis No explanation Incorrect explanation Irrelevant explanation
1	Accurately indicates that hypothesis was supported or not supported, and simply lists data without an explanation
2	Accurately indicates that hypothesis was supported or not supported, and Gives basic explanation with direction of relationship but no data to support the relationship. May have data on one variable but not the corresponding variable May use lay terms instead of science terms, but direction of relationship is accurate
3	Accurately indicates that hypothesis was supported or not supported, and Uses data to support relationship explanations for both variables involved May have additional incorrect data

#### Classroom videos

To understand how the teachers scaffolded students as the support in the material scaffolds faded, we viewed the 50 videos of whole-class discussions. We were particularly interested in how teachers adjusted their support as the static, uniform scaffolds embedded in the instructional materials faded. With this fading, teachers had opportunities to respond to students’ needs by adding or adjusting support so that students could learn and succeed.

We used deductive and inductive procedures to create a coding scheme (see Table 3) that captured the teachers’ scaffolding moves (Derry et al. 2010). We began with a priori expected codes for teachers’ scaffolding moves that were deductively based on prior research: monitoring understanding, instructional moves for support, and handover to independence (Smit et al. 2012; van de Pol et al. 2010). We then inductively refined these codes by watching the videos to identify specific scaffolding moves that characterized each teacher’s approach to instruction. This resulted in four primary coding categories: monitoring understanding, adaptive use of instructional materials, handover to independence, and static support. The first three categories reflect the responsive characteristic of scaffolding because they capture the teachers’ efforts to assess and respond to students’ current understanding (Puntambekar and Kolodner 2005; Smit et al. 2012; van de Pol et al. 2010). Teachers can respond by adapting and complementing the support in materials to meet students’ needs and eventually give students more responsibility for their learning when students are ready. In contrast, the category of static support reflected a lack of dynamic change and alignment with students’ understanding in teacher’s scaffolding. We noticed from our data that the teachers could monitor understanding of individual students by circulating the classroom to talk to individuals, or they could monitor understanding of the whole class by asking questions directed at all students. Thus, we distinguished between monitoring individual or whole-class understanding in our coding scheme. To characterize the adaptive use of instructional materials, we inductively identified three secondary codes that described specific instructional moves that emerged as we watched the videos: peer idea-sharing, modeling, and extending materials. Peer idea-sharing involved teachers providing support by encouraging students to discuss their ideas and explanations with each other. Modeling involved teachers demonstrating or partially completing a task that was just beyond students’ level of understanding. Extending materials described ways that teachers used the instructional materials in innovative ways beyond their original purpose, including creating shared visuals and supplementary activities to promote discussions and conceptual understanding. Teachers integrated these moves when students struggled with a question or concept, indicating that teachers understood that students needed more support.

**Table 3:** Coding protocol of the teachers’ scaffolding moves

Primary code	Secondary code	Description	Example
Monitoring Understanding	Individual monitoring Whole-class monitoring	Monitoring understanding allows teachers to evaluate students’ current understanding at the individual and/or whole-class level. This may help teachers provide responsive support. The teacher may ask questions to check for conceptual understanding, elicit ideas, or reveal science practices	“Do you have any reasoning why?” “Who else had car mass as a control?”
Adaptive use of instructional materials	Peer idea-sharing	The teacher provides adaptive support by encouraging discussion among peers to check for conceptual understanding through explanation	“I want you to share it with another person to see if your hypothesis makes sense and your reasoning as well”
	Modeling	The teacher provides adaptive support by modeling or completing portions of a question or task that are just beyond the students’ level of understanding. The teacher uses this challenge as an opportunity to demonstrate process and/or reasoning skills	“I’m going to pull up the simulation so we can take a look at it and know what we’re doing on our computers today”
	Extending materials	The teacher provides adaptive support by using the instructional materials beyond their intended purpose, such as by projecting and reviewing data tables as shared visuals for discussion	“I’m going to put the big paper on the board so we can look at the data here”
Handover to Independence	–	The instructor provides responsive support by allowing students to demonstrate competency by fading support and allowing students to complete tasks independently. By fading support, the teacher can check if students are ready for transfer of responsibility for learning or if students need additional temporary support	“So *you* fill in your hypothesis right now and write why you think this is true. Make sure your statement makes sense”
Static Support	–	The instructor provides “blanket” support for the whole class or completes an answer or task for the student without engaging student reasoning (i.e., “tells the student what to do.”)	“Let me write it down before you do. So what we are trying to get at is the rate at which mass increased…I just had the sentence. Again, you might want to wait until I finish”

We segmented the whole-class discussion videos into two-minute intervals (Borko et al. 2008) for consistency of coding and adequate time for development of distinct instructional moves. We coded for the presence of instructional moves within each segment. The first and second authors coded 20% of the data and achieved “substantial agreement” with a Cohen’s kappa of .78 for inter-rater reliability (Stemler 2001); disagreements were resolved through discussion and the remaining video data was divided between the two authors and coded independently. We then calculated the proportion of scaffolding moves for each video by dividing the total codes by the number of two-minute segments in each video, to standardize frequencies across unequal lesson lengths.

To analyze differences in scaffolding moves between the two teachers, we compared the proportions of each teacher’s scaffolding moves over the three levels of support (High, Medium, and Low) using a factorial ANOVA, with teacher and the level of support as independent factors. Our unit of analysis was the proportion of each of the scaffolding moves. We examined both interaction and main effects models. As we conducted ANOVA tests in RStudio, we used the *car* package to obtain Type III sum of squares.

## Results

The following sections detail our findings about differences between the two classrooms in terms of students’ learning, including the Report Out activities, and teachers’ scaffolding moves during whole-class discussions.

### Differences in student performance

#### Test scores

We compared students’ performance on the physics conceptual test and the science practices test at the beginning and end of the unit. Table 4 shows descriptive statistics for pre- and post-tests. Pre-post comparisons of both tests indicated that Mrs. Lewis’s and Mr. Green’s students learned a significant amount of physics content and science practices by the end of the unit. A mixed-model ANOVA with teacher and time as factors (see Table 5) showed that both teachers’ students (subjects in Table 5) improved on the physics conceptual test from pre- to post-test, as shown by the significant main effect of time (*F*(1,70) = 61.49, *p* < .001). There was also a significant interaction between teacher and time (*F*(1,70) = 11.01, *p* = .001). While Mrs. Lewis’s students had lower scores on the pre-test than Mr. Green’s students, Mrs. Lewis’s students overcame this disadvantage and these differences were no longer present on the post-test. Both classes also significantly improved on the science practices test from pre- to post-test, as shown again by the significant main effect of time (*F*(1,70) = 72.16, *p* < .001); the teacher main effect and interaction between teacher and time were not significant.

**Table 4:** Descriptive statistics for students’ performance on physics conceptual test and science practices test

Teacher	*n*	Pre-test		Post-test	
		M	SD	M	SD
Test					
Physics conceptual					
Mr. Green	44	14.70	2.50	16.98	3.87
Mrs. Lewis	28	12.00	3.33	17.25	3.42
Science practices					
Mr. Green	44	13.36	5.72	16.67	5.33
Mrs. Lewis	28	12.16	4.75	16.58	3.65

**Table 5:** Mixed-model ANOVA summaries for students’ performance on physics conceptual test and science practices test

Source	*df*	*SS*	*MS*	*F*	*p*
Physics conceptual test					
Between subjects	71	1095.99	50.60	3.39	.070
Teacher	1	50.60	14.93		
Subjects within teacher	70	1045.39			
Within subjects	72	981.50			
Time	1	423.67	423.67	61.49	< .001
Teacher × time	1	75.84	75.84	11.01	.001
Time × subjects within teacher	70	481.99	6.89		
Total	143	2077.49			
Science practices test					
Between subjects	71	3120.22			
Teacher	1	14.18	14.18	0.32	.573
Subjects within teacher	70	3106.04	44.37		
Within subjects	72	1004.50			
Time	1	504.38	504.38	72.16	< .001
Teacher × time	1	10.49	10.49	1.50	.225
Time × subjects within teacher	70	489.63	6.99		
Total	143	4124.72			

#### Report Out activities

We analyzed students’ Report Out responses throughout the curriculum based on the scoring scheme in Table 2. We performed a repeated-measures ANCOVA with teacher as the between-subjects factor, material scaffolding level as the within-subjects factor, and pre-test scores on the physics conceptual test and science practices test as covariates to account for students’ prior knowledge. We included pre-test scores to account for differential benefits of scaffolds for students with different prior knowledge (Belland et al. 2015).

**Fig. 1:**
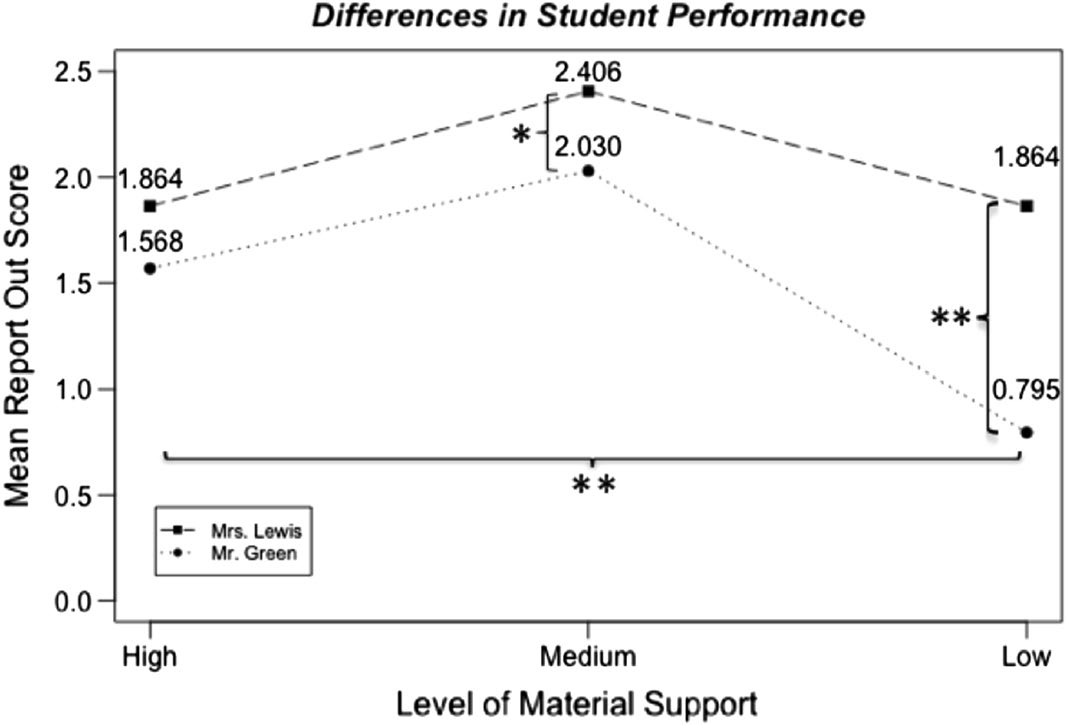
Differences in students’ performance on Report Out activities as material support faded throughout the curriculum, by teacher. Note: *significant at *p* < .05 and **significant at *p* < .001

Our analyses revealed a significant interaction between the instructor and the material scaffolding level (High, Medium, or Low Material Support) (*F*(2,208) = 8.923, *p* < .001). We conducted pairwise comparisons to investigate this difference between teachers, using *t*-tests and a Holm–Bonferroni correction. Students’ Report Out scores were not significantly different across teachers for the experiments with High Material Support (*t*(66.84) = 1.92, *p* = .059). However, Mrs. Lewis’s students performed significantly better than Mr. Green’s students on the Report Out activities in the experiments with Medium Material Support (*t*(54.37) = 2.81, *p *= .013) and Low Material Support (*t*(55.27) = 6.23, *p* < .001). These results are shown in Fig. 1.

We also conducted pairwise comparisons for differences across scaffolding levels for each teacher, using repeated measures *t*-tests with a Holm–Bonferroni correction to see how students performed over time. These comparisons gave us information about changes in student performance as material scaffolds faded and content became more challenging. Mrs. Lewis’s students’ performance on Report Out activities significantly improved as support faded from High to Medium (*t*(27) = − 4.77, *p* < .001), but then returned to the initial performance level as support faded from Medium to Low (*t*(27) = 4.41, *p* < .001). Thus, there was no difference in students’ performance between the High and Low scaffolds (*t*(27) = − 0.006, *p* = .996). Students in Mr. Green’s classroom showed similar improvement on Report Out activities as support faded from High to Medium (*t*(43) = − 3.80, *p* < .001). However, his students’ performance declined significantly as support faded from Medium to Low (*t*(43) = 10.31, *p* < .001), resulting in a significant decline in performance across the entire unit (*t*(43) = 6.19, *p* < .001). Together, the ANCOVA and pairwise comparisons showed that as the instructional materials faded in support, Mrs. Lewis’s students performed significantly better than Mr. Green’s students on the Report Out activities, as shown in Fig. 1.

### Differences in teachers’ scaffolding moves

Based on our findings that Mr. Green’s students did not perform as well as Mrs. Lewis’s students on Report Out activities as support faded in the instructional materials over time, we wanted to understand how teachers’ scaffolding moves over the unit may have impacted students’ performance. Our aim was to understand whether students needed additional support when the support in the Scientist’s Journals faded, and whether teachers adjusted the scaffolding they provided when support in the journals faded. To do this, we compared scaffolding moves between the two teachers over time (as material scaffolds faded in support). As described earlier, we coded responsive scaffolding moves as individual or whole-class monitoring, peer idea-sharing, modeling, extending materials, and handover to independence. We also coded for moves indicating static support provided by the teacher.

**Fig. 2:**
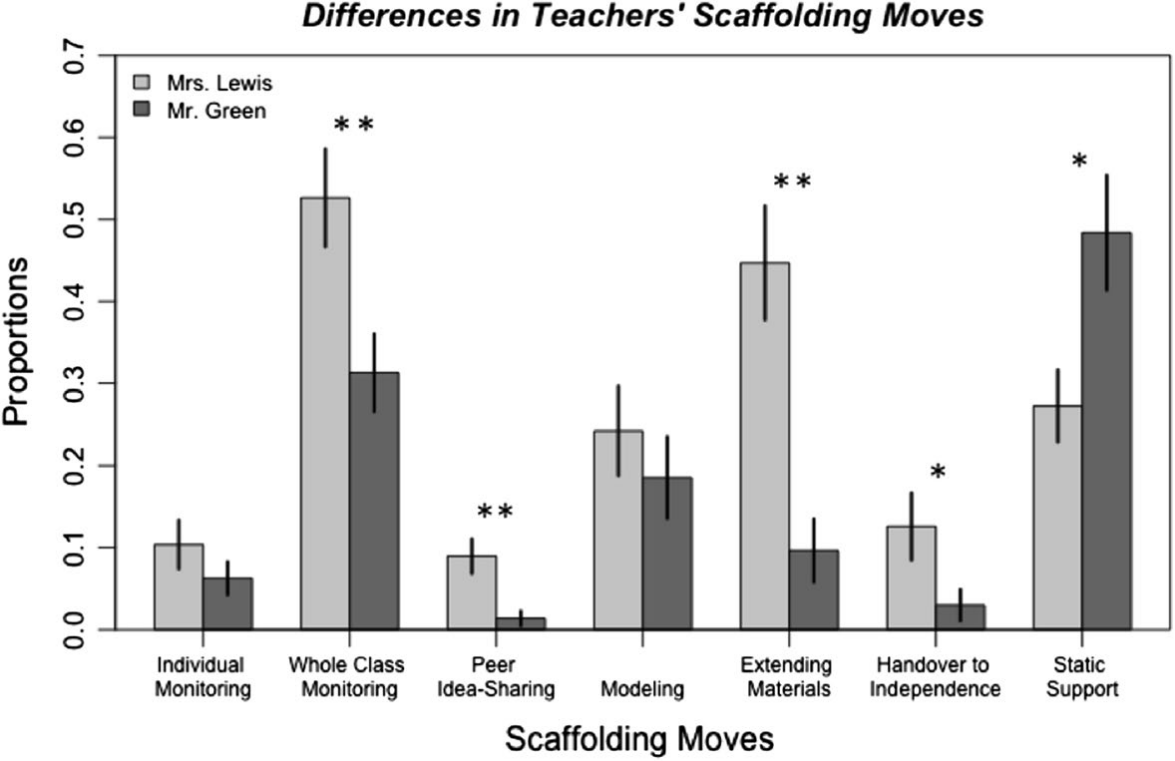
Differences in instructional moves between teachers. Note: *significant at *p* < .05 and **significant at *p* < .01

We conducted factorial ANOVAs for each scaffolding move, with teacher and level of support (High, Medium, and Low) as independent factors. We tested both interaction and main effects models. We found several significant main effects for teachers. The level of support was not significant in any of the main effect models, and none of the interaction models were significant. This means that the teachers’ scaffolding did not significantly differ over time. Thus, we report only differences due to a main effect for teachers below. Figure 2 shows differences in scaffolding moves among the two teachers.

#### Monitoring

We found a significant main effect for teacher (*F*(1,46) = 7.24, *p* < .001) in whole-class monitoring. Mrs. Lewis engaged in whole-class monitoring an average of 52.6% of the time, compared to 31.3% for Mr. Green. There was no significant main effect for teacher in individual monitoring (*F*(1,46) = 1.19, *p* = .280).

#### Adaptive use of instructional materials

We found a significant main effect for teacher (*F*(1,46) = 10.15, *p* = .003) in prompting peer idea-sharing. Mrs. Lewis encouraged peer idea-sharing an average of 9.0% of the time, compared to 1.4% for Mr. Green. We also found a significant main effect for teacher (*F*(1,46) = 17.67, *p* < .001) in extending materials. Mrs. Lewis extended the support embedded in materials an average of 44.7% of the time, compared to 9.6% for Mr. Green. There was no significant main effect for teacher in modeling (*F*(1,46) = 0.75, *p* = .391).

#### Handover to independence

We found a significant effect for teacher (*F*(1,46) = 4.58, *p* = .038) in moves that encouraged more independence (i.e., fading). Mrs. Lewis encouraged handover an average of 12.6% of the time, compared to 3.0% for Mr. Green.

#### Static support

We found a significant effect for teacher (*F*(1,46) = 4.58, *p* = .038) in moves that repeated the static support embedded in the materials. Mr. Green replicated the support in the students’ journal an average of 48.4% of the time, compared to 27.3% for Mrs. Lewis.

#### Summary

We found that Mrs. Lewis engaged in more whole-class monitoring and adapted materials by encouraging peer idea-sharing and extending the material-based support than Mr. Green. Mrs. Lewis was also engaged in moves that encouraged handover to independence more often than Mr. Green. In contrast, Mr. Green provided support that was redundant to support embedded in the materials.

### Qualitative description of differences in scaffolding moves

To illustrate differences in the teachers’ scaffolding moves, we identified examples that characterized each teacher’s instructional approach. These examples show how (a) Mrs. Lewis provided more responsive support during the unit that extended and complemented the material scaffolds; and (b) Mr. Green provided more static support that replicated the material scaffolds.

#### Mrs. Lewis: responsive and complementary support

The following excerpts illustrate Mrs. Lewis’s scaffolding moves and how she provided responsive support for her students as the instructional materials faded, complementing the static material scaffolds. In Mrs. Lewis’s teaching, we see evidence of whole-class monitoring, extending the support in the instructional materials, modeling, and eventual handover to independence. The excerpt in Table 6 shows as example of how Mrs. Lewis frequently monitored students’ understanding and utilized the whiteboard to extend materials to help her students organize their data and identify conceptual relationships. She reviewed the first three simulation experiments with her class by creating a chart (based on students’ responses) of the independent, controlled, and dependent variables for each experiment. Afterward, she provided a worksheet that she had designed for students to describe key science connections from each experiment. She demonstrated how to use this worksheet to write relationships between variables and then encouraged students to work independently on the remaining questions.

**Table 6:** Example 1 of Mrs. Lewis’s scaffolding moves with codes

Speaker	Speech	Codes
Mrs. Lewis:	**Before I send you into groups, we’re going to talk about what we’ve done already just to refresh our memories.** So we’ve done three different simulations, and in each one we’ve changed something. Who can tell us one thing that we’ve changed in some of our simulations? Maria?	Whole-class monitoring
Maria:	The mass of the car	
Mrs. Lewis:	And that was the first one that we did. We changed the mass. ***[writes “mass” on whiteboard]*** What else, Jack?	Extending materials
Jack:	The height	
Mrs. Lewis:	We changed the height, and that was the second thing that we changed. ***[writes “height” on whiteboard in separate column]*** And what was the last thing that we did? Marcus?	Extending materials
Marcus:	Friction	
Mrs. Lewis:	We changed friction. ***[writes “friction” on whiteboard in separate column]*** Okay, so when we changed the mass of the car, what were some things that we left alone? What did we not change? Julia?	Extending materials
Julia:	The friction	
Mrs. Lewis:	We left the friction the same, to zero. ***[writes “friction” underneath “mass”]*** What else did we leave alone? Stephen? …	Extending materials
	*[Mrs. Lewis continues to ask students about controlled variables in each simulation experiment,* ***listing controlled variables underneath the independent variables*** *as students respond. She does the same for dependent variables in each experiment. Mrs. Lewis then prepares the class to do group work and offers use of the simulation if needed.]*	Extending materials
Mrs. Lewis:	**And the other thing that I’m going to have you do, to hopefully help you see some of these connections, is a sheet that lays it all out for you.** Go ahead and put your name on this, and we’re going to do one of these together. *[passes out worksheets]*	Extending materials
Mrs. Lewis:	So this is called, “What connections am I making?” Think about the simulations you have been doing with your roller coaster. What are you learning and noticing? I broke this up into chunks based on the three simulations that we’ve done. The first chunk–how does mass affect applied force, work, potential energy–I’m going to have you turn back to page 21, and **we will answer the first one together. Take a look at your data chart. What does changing the mass do to the applied force? It’s probably a good idea to use words like increase, decrease, stay the same**, since those are the terms we were using in our hypotheses and our Report Out questions. So when you look at your data, what do you see? Marcus, what do you notice?	Modeling
Marcus:	Applied force increases	
Mrs. Lewis:	The applied force increases as—what is the mass doing though?	
Marcus:	Increasing	
Mrs. Lewis:	The mass is increasing also? Take a look at your chart and give a thumbs-up if you also see “as mass increases, applied force increases.” Who else notices that from page 21? Does anyone not see that? Is anyone seeing something different? If you do, you should probably re-check your data, because that’s what should be happening. **So for the first one, we’re all going to write this: “As the mass increased, the applied force also increased”*[writes on whiteboard]*** **So this is the format that I want you to use when you’re answering the questions** It may get a little repetitive, I understand that, but at least **we’re being very detailed. We’re not just saying, “It went up. It changed.” We want to know what “it” is. We’re saying, “As the mass increased, the applied force also increased.”** **You might have to write the same thing over a few times, but at least you’ll have a very clear idea of how one thing affects another**	Modeling Handover to independence Modeling Handover to independence

Bolded statements in Tables 6, 7, 8 and 9 correspond to the codes on the right

Mrs. Lewis’s framing of the activity in this example as a review of what students had learned clearly established her intention to monitor understanding. This excerpt also exemplifies how she extended the material-based support in the curriculum by creating a chart to organize students’ ideas on the whiteboard to support making connections between variables. The worksheet she created further extended this support for understanding relationships and also allowed her to model how to explicitly link variables and write relationships. Importantly, we see that this instance of monitoring understanding and providing support in response culminated in Mrs. Lewis handing over responsibility to her students to work independently, characterizing the key aspects of scaffolding.

The excerpt in Table 7 (which occurred near the end of the unit) provides additional evidence of how Mrs. Lewis extended material-based support and illustrates how she used peer idea-sharing to support students. As a reminder, the Scientist’s Journals introduced fading of support by (a) re-structuring the hypothesis-writing activities to open-ended statements, and (b) providing blank data charts, versus partially completed charts. We were interested in how the teachers helped students work through this fading of support as students engaged in the Report Out activities. In this example, Mrs. Lewis provided sample Report Out statements and encouraged students to critique them. She asked students to discuss their ideas with a partner and then modeled ideal Report Out statements based on students’ ideas.

**Table 7:** Example 2 of Mrs. Lewis’s scaffolding moves with codes

Speaker	Speech	Codes
	*[The projector displays: “What are the pros/cons of this “report*-*out”? My hypothesis was confirmed. The experiment showed that as it increased, the net force on the riders did too. Here are some examples from my data: Trial 1*–*12* *N, 1.5; Trial 2*–*27.5* *N, 3.”]*	
Mrs. Lewis:	I have a couple of warm-ups for you. I will be scrolling this down *[referring to projector*]. There are three of these. **I’ve made some Report Outs for you,** and **I want you to discuss with someone near you**, what are some things that are good about these Report Outs, and what are some things that could probably be done a little bit better, more scientifically made a little bit more clear. **Go ahead and talk about this one**, **then we’ll talk about it as a whole group**, and then we’ll move on to the next one. *[Students discuss the quality of the Report Out projected on the screen]*	Extending materials Modeling Peer idea-sharing Peer idea-sharing Whole-class monitoring
Mrs. Lewis:	Let’s talk about this together. Good things? Things that could be better? What did you notice? What do you see? What sort of advice would you give the person who wrote this Report Out? Nate?	
Nate:	We don’t know what the numbers are representing	
Mrs. Lewis:	Why?	
Nate:	It doesn’t say friction or whatever. [inaudible]	
Mrs. Lewis:	So down here, the data–we know we have 12 Newtons for something, maybe it’s telling us force. **Then we have 1.5 and 3. So we don’t really know what those numbers are telling us. I understand that friction does not have a label, but you should still indicate level of friction. So, that’s a good thing to pick out. We need to put labels. We need to make sure we’re clear with what our data is.** Sasha, what else? What other advice do you have?	Modeling
	*[Mrs. Lewis continues and repeats this activity with two more sample Report Out statements.]*	

This excerpt showed how Mrs. Lewis complemented the fading of the Scientist’s Journal by providing additional examples that extended beyond the support in the curriculum and by modeling ideal responses. This excerpt also exemplifies how she leveraged peer idea-sharing in combination with the instructional materials. She created an opportunity for students to share their ideas with the whole class by creating a different opportunity to share with peers first.

#### Mr. Green: static and replicated support

The following excerpts came from Mr. Green’s classroom and illustrate how Mr. Green missed opportunities to provide additional scaffolding needed by students as support in the instructional materials faded. The excerpt in Table 8 demonstrates how Mr. Green simply replicated the structure of the static, uniform scaffolds found in the instructional materials. He asked students to fill in sentences about the physics relationships they investigated in their recent experiments to help them to construct reports of their results. These sentences were consistent with the fill-in-the-blank hypotheses provided in the instructional materials in the phase with Medium Material Support; students only needed to write in the words “increase”, “decrease”, or “stays the same” to complete the hypotheses. Mr. Green emphasized the fill-in-the-blank nature of the activity by telling students that they did not need to write complete sentences, and he continued this emphasis when he called on students to share their one-word answers. He evaluated their answers as correct or incorrect but did not further explain or elaborate on relationships, even when his students may have needed further explanation. When one student (Carl) gave an incorrect answer, Mr. Green told him that his data was incorrect but did not respond with further support that aligned with this student’s understanding.

**Table 8:** Example 1 of Mr. Green’s scaffolding moves with codes

Speaker	Speech	Code
Mr. Green:	**I want you to answer these questions with either “it increased”, or “it decreased”, or “it stayed the same”**	Static support
Max:	That’s all we have to say?	
Mr. Green:	Yes, you don’t need to write any complete sentences at this point. Right now we are just trying to grasp that initial understanding of when you are reading your data	
	*[Students complete worksheet.]*	
Mr. Green:	**Let’s go over what you are thinking here.** We have 10 answers, so I’m hoping to get 10 people involved. And I may not just call on the kids that raise their hands. So you need to be available so I know you are paying attention. Joe number 1, when we compare mass to applied force?	Whole-class monitoring
Joe:	Increase	
Mr. Green:	**So your data should have showed that it increased. Right**. Mass versus work, um, let’s see. Trevor? What should have happened? Mass versus work	Static support
Trevor:	Increased	
Mr. Green:	Increased. Okay, Matthew, mass versus potential energy?	
Matthew:	Increase	
Mr. Green:	**All right, so it’s increase for all three.** Carl, for height and force?	Static support
Carl:	Decrease	
Mr. Green:	**It should not have decreased.** Now this is one that we talked about. Remember just now, the three of you, **I told you that your data was incorrect for the middle and for the third.** Allison?	Static support Static support
Allison:	Stayed the same	
Mr. Green:	**Should have stayed the same. In this particular case your applied force should have stayed the same**	Static support

This example showed how Mr. Green did not alter the scaffolded structure of the fill-in-the-blank hypotheses to align with the needs of his students. He maintained and replicated the structure of the task and only assessed his students’ ability to provide a correct, one-word answer during a whole-class discussion structured by initiate-response-evaluate dialogue (Mehan 1979). By adhering to the structure of the task, Mr. Green’s monitoring of student understanding did not assess deeper conceptual understanding. This “shallow” monitoring for understanding and hesitance to adapt the task to his students’ needs resulted in greater difficulty later in the unit as the hypothesis tasks became more challenging.

The excerpt in Table 9 further illustrates Mr. Green’s replication of static support provided in the instructional materials, as compared to responsive support. This excerpt took place during the last experiment of the unit, in which the data table was intentionally open-ended so students could demonstrate competency in science practices. Mr. Green held up a scientist’s journal and *told* students how to fill out the data chart in preparation for their next experiment, rather than taking time for students to attempt the data table on their own. It is unclear whether students could have completed this activity on their own as they were not given the opportunity. Instead, Mr. Green told them which variables were held constant in the experiment and gave instructions for what to write in the table.

**Table 9:** Example 2 of Mr. Green’s scaffolding moves with codes

Speaker	Speech	Code
Mr. Green:	Now on this page we are missing constants. You know how normally [the experiment page] gives you constants listed outside of the box and so forth? There is nothing listed here. **So let’s list them along the side here. We need friction. So you want to write the word friction and draw a blank.** Ok? And we need drop height; wait not drop height, hill height. Hill height, and we need shape. **So somewhere you need to list those, because this is what you are starting with. This is what you are using for all three of these. The reason we are not putting in ID height or mass, those are going to be located within your trials.** Ok? When you get done, you have questions to answer back here	Static support Static support

This excerpt illustrates how Mr. Green relied predominantly on replicating static scaffolds in the instructional materials rather than providing adaptive, verbal scaffolding that was responsive to students’ needs. In this case, Mr. Green could have modeled how to think about variables in the data table or encouraged peers to discuss different kinds of variables. Instead, Mr. Green gave exact instructions for completing the task and thus missed opportunities to monitor students’ understanding at the end of the unit. This type of instruction does not facilitate handover to independence, which relies on calibrated fading of support as students take on greater responsibility for their learning.

## Discussion

Distributing support across multiple tools, resources, and agents can address the challenge of supporting multiple students and their diverse needs in a classroom (Luckin 2010; Puntambekar and Kolodner 2005; Tabak 2004), especially for students solving complex, ill-structured problems (Hmelo-Silver and Barrows 2006). But, when multiple scaffolds are used, we need to understand how they work together, to inform both the design of innovative instructional materials and guidance for practitioners. Our aims in this study were to understand (a) how material scaffolds with gradually faded support (through changes in structure of prompts and data tables) helped students to solve complex problems in the classroom, and (b) how the relationship between scaffolding provided by the teacher and scaffolding in the paper-and-pencil tool related to students’ learning. Our main findings were as follows:

- When material scaffolds faded from High to Medium support, students in Mrs. Lewis’s classes and Mr. Green’s classes improved their performance on the Report Out activities, but Mrs. Lewis’s students significantly outperformed Mr. Green’s.
- When material scaffolds faded from Medium to Low support, students in both teacher’s classes showed poorer performance on the Report Out activities, and Mr. Green’s students did significantly worse than Mrs. Lewis’s.
- Mrs. Lewis provided more responsive support for students, while Mr. Green provided more static support. She frequently used strategies such as monitoring understanding, extending support in the instructional materials, and handing over independence to students.
- Mrs. Lewis’s responsive support complemented the support built into the instructional materials, while Mr. Green’s support replicated this support. Essentially, Mrs. Lewis elaborated on the material support in response to students’ understanding, while Mr. Green did not elaborate on the material support.
- The complementary combination of responsive teacher scaffolding and gradually fading material scaffolds was more beneficial for students’ learning.

We discuss our findings about differences in students’ performance with respect to (a) the fading of the material scaffolds and (b) the interplay between the teacher and material scaffolding to answer each of our research questions.

### Fading in material scaffolds

In order to design scaffolding for classrooms that can help individual students within their own ZPDs, we must understand both the tasks being scaffolded and students’ general range of understanding. Wood et al. (1976) original description of scaffolding suggested that ‘‘theory of the task’’ and ‘‘theory of the tutee’’ were crucial to providing effective scaffolding. Therefore, the first step for designing scaffolding for the classroom is to understand the range of knowledge and skills students typically come with, so that instruction can be planned based on multiple ZPDs. Scaffolds embedded in instructional materials are often based on guidelines and frameworks informed by the difficulties that students have, the kind of support students might need, and the kinds of challenges students can successfully tackle (Quintana et al. 2004; Reiser 2004; Saye and Brush 2002).

In our study, the Scientist’s Journals were based on our previous work on supporting students to solve design problems. One of our goals was to investigate how to integrate fading into paper-and-pencil tools by decreasing the level of structure in prompts and data tables. But, as discussed by Lajoie (2005), it is challenging to make decisions about when and how to fade support, especially in static materials. Because we incorporated multiple opportunities for students to engage in the practice of science, we gradually decreased the level of material support in later parts of the unit, by which time students had several opportunities to engage in practices such as hypothesis writing, collecting and recording data, and reporting that data. Our aim was to examine whether the level of fading, which was relatively gradual and involved only three levels, would affect students’ responses in their Report-Outs. It is important to note that even for activities with Low Support, there was some support for students; that is, support was not totally absent in any part of the unit.

We found that when material support decreased from High to Medium, students in Mrs. Lewis’s and Mr. Green’s classes improved on reporting and explaining results from their experiments in the Report Out activities. Here, fading the material support benefited students. This aligns with prior findings that fading support in instructional materials is more helpful for students than maintaining constant support (McNeill and Krajcik 2009; McNeill et al. 2006). However, we found that when material support faded from Medium to Low, students’ performance on the Report Out decreased for both teachers. Mrs. Lewis’s students returned to their initial level of performance (i.e., same performance in High and Low), but Mr. Green’s students performed significantly worse during the Low Material Support experiments than the High Material Support experiments. This continued fading of support in the Scientist’s Journals seemed to ask too much of students, especially Mr. Green’s students. Thus, even though students had some level of support and previous opportunities to engage in science practices, the fading of support did not effectively support students’ independent work. In this distributed scaffolding context, our analysis of the types of scaffolding provided by the two teachers helped us understand these results.

### Interplay between teacher and material scaffolds

#### Repetition, extension, and complementarity

A key difference we found between Mrs. Lewis’s and Mr. Green’s approach to scaffolding was the more responsive nature of Mrs. Lewis’s support. She frequently monitored students’ understanding, provided additional support as needed, and included opportunities to hand over responsibility to students for their learning. In contrast, Mr. Green tended to replicate the support found in the instructional materials, rather than monitor his students’ understanding and their reactions to the level of support provided in the materials. This means that when material support faded, Mr. Green’s support faded too, regardless of students’ needs. Thus, he did not adapt his support accordingly. As effective scaffolding necessitates contingency (Koole and Elbers 2014; van de Pol et al. 2010, 2014), Mr. Green’s repetitive–rather than adaptive–support resulted in a lack of responsiveness and minimal handover to independence.

The scaffolding embedded in the Scientist’s Journals may have faded too quickly for Mr. Green’s students, and his limited monitoring of students’ understanding and lack of responsive support may explain why his students’ performance decreased as the unit became more open-ended. Mr. Green’s tendencies to give exact instructions to the whole class at the outset of activities and to focus on single correct answers were sufficient in helping students to work through more structured experiments that included partially completed hypothesis prompts and data tables at the beginning and middle of the unit (i.e., High and Medium Material Support). However, as students answered more open-ended questions at the end of the unit (i.e., Low Material Support), these strategies did not sufficiently complement the fading of support in the instructional materials or promote independent reasoning. By replicating the material scaffolding, Mr. Green may have missed opportunities to diagnose students’ understanding and provide the kind of dynamic support that characterizes scaffolding (Ge and Land 2004; Puntambekar and Hübscher 2005; Saye and Brush 2002; van de Pol et al. 2010).

Mrs. Lewis’s responsive support, on the other hand, *complemented* the gradually faded support in the instructional materials. She extended the support in the instructional materials to help students analyze patterns in their data; identify relationships between variables; make connections between key science concepts; and use data to support claims. Our qualitative results exemplify how Mrs. Lewis monitored students’ understanding and adapted the instructional materials to provide support that differed from–and complemented–what was built into the Scientist’s Journals. As the material support faded, Mrs. Lewis continued to provide responsive support that addressed students’ needs. In an ideal system of distributed scaffolding, as material scaffolds fade, the teacher monitors students’ understanding to check if students are ready for that fading and provides complementary support as needed (Puntambekar et al. 2007). If material scaffolds fade too quickly for students, teachers may need to continue providing the prior level of support to complement this fading. While we cannot make claims about Mrs. Lewis’s pedagogical decision-making from our data, her frequent monitoring of students’ understanding suggests that she was aware of her students’ needs and may have realized that students needed additional support as the material scaffolds faded. By offering an additional layer of scaffolding for students that was different from the static form of support found in the instructional materials, Mrs. Lewis’s scaffolding complemented the material scaffolds when they were not sufficient for students. Overall, our findings support and build on the notion that increased responsiveness in teachers’ scaffolding can mitigate students’ difficulties with material scaffolds (Belland et al. 2015; Ge and Land 2004; Saye and Brush 2002) and provide important supplementary support to material scaffolds by dynamically catering to students’ needs (Songer et al. 2012).

#### Responsiveness and contingent support

When designing the paper-and-pencil tool to support students’ complex problem-solving, we were able to incorporate fading by estimating students’ anticipated difficulties. However, incorporating fading into a static tool creates some risk of struggle for students, especially students with lower prior knowledge. Our findings also support the idea that responsive, complementary support from teachers is critical for students with low prior knowledge (Azevedo et al. 2016; Belland 2010, 2011; Belland et al. 2015; Raes et al. 2012). The fact that Mrs. Lewis’s students overcame a conceptual deficit compared to Mr. Green’s students, as they began with significantly lower prior knowledge and then outperformed Mr. Green’s students on the Report Out activities, suggests that Mrs. Lewis’s responsive scaffolding had a major impact on students’ learning. Given that the material scaffolds were identical for both groups of students, the differences in teachers’ scaffolding moves seem to explain this difference. A teacher or human tutor may provide additional just-in-time scaffolding when a learning environment does not meet students’ needs (Azevedo et al. 2016; Kim and Hannafin 2011; Raes et al. 2012), but the quality of this scaffolding is key to students’ learning.

The key idea behind scaffolding is that students eventually internalize strategies for completing a task. As explained by Wood et al. (1976), what is important about scaffolding that is contingent on the learner’s progress is that the student not only learns how to complete a specific task, but also abstracts the strategies used to complete the activity. The teachers in our study did not necessarily help students understand the reasoning for each activity so that students could later function independently as material support faded. Yet, Mrs. Lewis did this to some extent, such as by modeling what the Report Out activities entailed, which might have helped students to internalize the reporting process over multiple experiments so that they could complete the Report Out activities on their own after the material support faded. While we had hoped to see greater transfer of responsibility for learning to students over the unit, Mrs. Lewis may have realized, through monitoring her students’ understanding, that they were not prepared for the fading of support embedded in the instructional materials. Her consistent, responsive scaffolding moves complemented the fading of material support in a way that Mr. Green’s replicative support did not and, thus, played a critical role in her students’ higher performance during the unit compared to Mr. Green, especially as the prompts and tables in the Scientists’ Journals became more open-ended.

#### Integrating multiple, distributed scaffolds

A central feature of distributed scaffolding is that the system of scaffolds allows students to take advantage of different forms of support as needed, often at multiple times over a unit. In previous work, we found that redundant scaffolds helped students as they engaged with the same content and practices over multiple resources and activities (Puntambekar and Kolodner 2005). But, this study showed that when material support was not optimal or faded too quickly for students’ understanding, mere redundancy did not help. Redundancy is not effective when students need more help than what is presented in the materials. Our findings help characterize an important feature of teacher scaffolding when multiple distributed scaffolds are present: that it should extend beyond and complement the support already built into the learning environment, rather than simply replicating existing support. By providing responsive support that complemented the support in the Scientist’s Journals, Mrs. Lewis was able to *add* another layer of scaffolding to the system of distributed scaffolds in her classroom.

Our findings highlight the importance of the participatory relationship between teachers and instructional materials in classrooms (Remillard 2005; Tabak and Reiser 1997). Material scaffolds can support students’ learning, but instructional materials often cannot stand alone in classrooms to support students with different needs and ZPDs. In alignment with prior research, relying solely on scaffolded instructional materials runs the risk of de-emphasizing the dynamic and calibrated support individual students need, because material scaffolds are often predefined and thus not adaptive to individual students’ understanding (McNeill et al. 2006; Puntambekar and Hübscher 2005). While fading of support is an important characteristic of scaffolded learning environments (Lajoie 2005; Pea 2004; Puntambekar and Hübscher 2005), our findings support the idea that fading of support in instructional materials can occur too quickly for some students and thus hurt their performance (as especially seen with Mr. Green’s students). Teacher scaffolding that complements the fading support in material scaffolds seems crucial for preventing this decrease in performance. Material scaffolds can fulfill an important role in addressing students’ needs (Luckin 1998; Reiser 2004), but our work further supports the idea that the ways in which teachers monitor and adapt material scaffolds is essential for student learning (Puntambekar et al. 2007).

## Conclusions and future directions

Distributed scaffolding allows students to take advantage of different forms of support from multiple tools and agents as needed (Luckin 2010; Puntambekar and Kolodner 2005; Tabak 2004). However, successfully capitalizing on distributed scaffolds may require a level of metacognitive awareness that students are not ready for (Puntambekar and Kolodner 2005). Teachers are in a position to monitor students’ needs with respect to the whole system of support and adapt support accordingly. Considering and anticipating what exactly a teacher can provide that other tools in a learning environment cannot is an important step that should not be overlooked and warrants further investigation. Our study showed that the complementarity between responsive scaffolding moves from the teacher and scaffolding embedded in instructional materials is important when implementing distributed scaffolding that effectively supports the wide range of students’ abilities and needs in the classroom. Understanding the effect of teachers’ scaffolding moves for mediating material scaffolds has implications for designing scaffolded learning environments and preparing teachers to successfully support students in their classrooms. Fading support in instructional materials can be beneficial for students, but fading support too quickly can hurt students’ performance. Acknowledging that material scaffolds can be useful but may not fade at an appropriate pace for all students draws attention to a need for better preparing teachers to understand whether instructional materials or tools are providing sufficient support for their students and how to complement this support when needed. Rather than thinking of teachers as another source of support, our findings suggest that teachers may be essential in calibrating the support provided by other tools and agents.

Our findings shed light on the complex relationship between multiple scaffolds and point to several areas for teacher professional development with respect to helping teachers understand how to monitor and adapt support. It will be important to help teachers understand their role in monitoring the effectiveness and fading of material scaffolds, as well as the importance of adapting material scaffolds to meet their individual students’ needs. Teachers are constantly navigating tensions between providing enough support for students to learn but not too much support that they are giving students answers and preventing students from learning to think independently. Teachers could be helped to understand how support has been built into tools or materials and how this support might exceed or fall short of the support their students need. Focusing on strategies for continually monitoring students’ understanding and why this is an essential step towards providing responsive support may help teachers make decisions about when and how to provide additional complementary support or let students work toward independence.

Our study also brings to the forefront the issue of fading. Pea (2004) makes a distinction between scaffolds-for-performance and scaffolds-with-fading. Scaffolds-for-performance (such as stairs) are the tools that cannot be taken away and function as props for specific activities. But scaffolds-with-fading are designed to help support learning and are intended to be taken away when the learner is able to perform the task unaided. Fading is a necessary component of scaffolding. Technology tools and written prompts may not be able to adjust to the individual learner and may not fade, but teachers can play an important role to achieve fading. In our study, teachers complemented the support provided by the written prompts that faded too quickly for some students. But we can envision situations where the teacher is responsible for making decisions about adjusting the level of support in material scaffolds. For example, the teacher could decide the quality and quantity of written prompts that students need, based on her assessment of students’ learning. Or she might be able to change the levels of support that are provided in software. This study leaves open questions about teachers’ awareness and intentional adjustment of support that could be investigated through work on how teachers could make such decisions. This requires us to think carefully about the design and integration of our tools in ways that will empower teachers rather than increasing their load (Dillenbourg et al. 2011; Dillenbourg 2013). We need more empirical studies to understand the interplay between material and social scaffolds, the tasks, skills, and knowledge that could be supported by each form of scaffold, and how the support could change over time and eventually fade.

## Figures and Tables

**Table 10:**
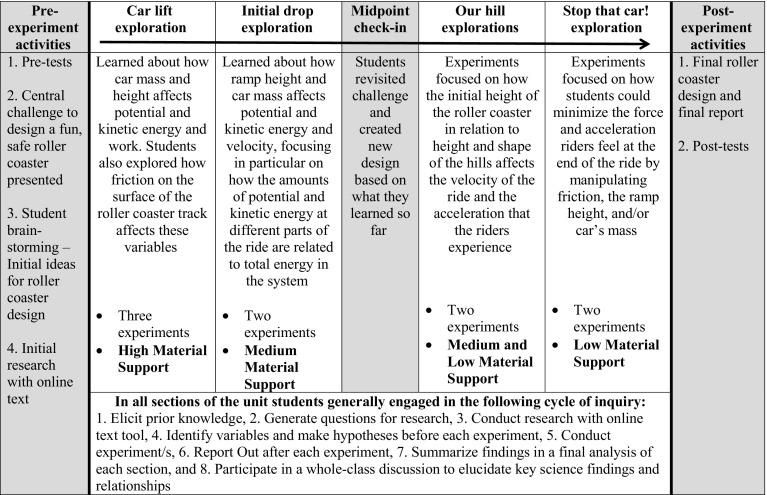
Aspects and sequence of the roller coaster unit

## References

[CR1] Azevedo R, Cromley JG, Moos DC, Greene JA, Winters FI (2011). Adaptive content and process scaffolding: A key to facilitating students’ self-regulated learning with hypermedia. Psychological Testing and Assessment Modeling.

[CR2] Azevedo R, Cromley JG, Winters FI, Moos DC, Greene JA (2005). Adaptive human scaffolding facilitates adolescents’ self-regulated learning with hypermedia. Instructional Science.

[CR3] Azevedo, R., Martin, S. A., Taub, M., Mudrick, N. V., Millar, G. C., & Grafsgaard, J. F. (2016). Are pedagogical agents’ external regulation effective in fostering learning with intelligent tutoring systems? In *International Conference on Intelligent Tutoring Systems* (pp. 197–207). Springer, Cham.

[CR4] Belland BR (2010). Portraits of middle school students constructing evidence-based arguments during problem-based learning: The impact of computer-based scaffolds. Educational Technology Research and Development.

[CR5] Belland BR (2011). Distributed cognition as a lens to understand the effects of scaffolds: The role of transfer of responsibility. Educational Psychology Review.

[CR6] Belland BR, Gu J, Armbrust S, Cook B (2015). Scaffolding argumentation about water quality: A mixed-method study in a rural middle school. Educational Technology Research and Development.

[CR7] Borko H, Jacobs J, Eiteljorg E, Pittman ME (2008). Video as a tool for fostering productive discussions in mathematics professional development. Teaching and Teacher Education.

[CR8] Brown AL, Ash D, Rutherford M, Nakaguwa K, Gordon A, Campione JC, Saloman G (1993). Distributed expertise in the classroom. Distributed cognition: Psychological and educational considerations.

[CR9] Bruner JS, Wertsch JV (1985). Vygotsky: A historical and conceptual perspective. Culture, communication, and cognition: Vygotskian perspectives.

[CR10] Chin C (2007). Teacher questioning in science classrooms: Approaches that stimulate productive thinking. Journal of Research in Science Teaching.

[CR11] Derry SJ, Pea RD, Barron B, Engle RA, Erickson F, Goldman R (2010). Conducting video research in the learning sciences: Guidance on selection, analysis, technology, and ethics. Journal of the Learning Sciences.

[CR12] Design-Based Research Collective (2003). Design-based research: An emerging paradigm for educational inquiry. Educational Researcher.

[CR13] Dillenbourg P (2013). Design for classroom orchestration. Computers & Education.

[CR14] Dillenbourg, P., Zufferey, G., Alavi, H., Jermann, P., Do-Lenh, S., Bonnard, Q., et al. (2011). Classroom orchestration: The third circle of usability. In *Proceedings of the 9th International Conference on Computer Supported Collaborative Learning*, Hong Kong, China, Vol. 1, (pp. 510–517).

[CR15] Epstein K, Hillegeist E (1990). Intelligent instructional systems: Teachers and computer-based intelligent tutoring systems. Educational Technology.

[CR16] Forman EA, Ramirez-DelToro V, Brown L, Passmore C (2017). Discursive strategies that foster an epistemic community for argument in a biology classroom. Learning and Instruction.

[CR17] Ge X, Land S (2004). A conceptual framework for scaffolding III-structured problem-solving processes using question prompts and peer interactions. Educational Technology Research and Development.

[CR18] Hmelo-Silver CE, Barrows HS (2006). Goals and strategies of a problem-based learning facilitator. Interdisciplinary Journal of Problem-based Learning.

[CR19] Kim MC, Hannafin MJ (2011). Scaffolding 6th graders’ problem solving in technology-enhanced science classrooms: A qualitative case study. Instructional Science.

[CR20] Koedinger KR, Corbett AT, Sawyer K (2006). Cognitive Tutors: Technology bringing learning science to the classroom. The Cambridge handbook of the learning sciences.

[CR21] Kolodner JL, Camp PJ, Crismond D, Fasse B, Gray J, Holbrook J (2003). Problem-based learning meets case-based reasoning in the middle-school science classroom: Putting learning by design (tm) into practice. Journal of the Learning Sciences.

[CR22] Koole T, Elbers E (2014). Responsiveness in teacher explanations: A conversation analytical perspective on scaffolding. Linguistics and Education.

[CR23] Kozulin A, Presseisen BZ (1995). Mediated learning experience and psychological tools-Vygotsky’s and Feurstein’s perspectives in a study of student learning. Educational Psychologist.

[CR24] Lajoie SP (2005). Extending the scaffolding metaphor. Instructional Science.

[CR25] Lajoie SP, Guerrera C, Munsie SD, Lavigne NC (2001). Constructing knowledge in the context of BioWorld. Instructional Science.

[CR26] Linn MC, Clark D, Slotta JD (2003). WISE design for knowledge integration. Science Education.

[CR27] Luckin, R. (1998). Knowledge construction in the zone of collaboration: Scaffolding the learner to productive interactivity. In A. Bruckman, M. Guzdial, J. Kolodner, & A. Ram (Eds.), *Proceedings of the International Conference of the Learning Sciences* (pp. 188–194). Charlottesville, VA: Association for the Advancement of Computing in Education.

[CR28] Luckin R (2010). *Re*-*designing learning contexts: Technology*-*rich, learner*-*centred ecologies*.

[CR29] Luckin R, Du Boulay B (1999). Ecolab: The development and evaluation of a Vygotskian design framework. International Journal of Artificial Intelligence in Education.

[CR30] McNeill KL, Krajcik J (2009). Synergy between teacher practices and curricular scaffolds to support students in using domain-specific and domain-general knowledge in writing arguments to explain phenomena. Journal of the Learning Sciences.

[CR31] McNeill KL, Lizotte DJ, Krajcik J, Marx RW (2006). Supporting students’ construction of scientific explanations by fading scaffolds in instructional materials. Journal of the Learning Sciences.

[CR32] Mehan H (1979). Learning lessons: Social organization in the classroom.

[CR33] Palincsar A, Brown AL (1984). Reciprocal teaching. Cognition and Instruction.

[CR34] Palincsar AS, Brown AL, Campione JC, Forman EA, Minick N, Stone CA (1993). First-grade dialogues for knowledge acquisition and use. Contexts for learning: Sociocultural dynamics in children’s development.

[CR35] Pea RD (2004). The social and technological dimensions of scaffolding and related theoretical concepts for learning, education, and human activity. Journal of the Learning Sciences.

[CR36] Puntambekar S, Hübscher R (2005). Tools for scaffolding students in a complex learning environment: What have we gained and what have we missed?. Educational Psychologist.

[CR37] Puntambekar, S., & Kolodner, J. L. (1998). The design diary: A tool to support students in learning science by design. In *Proceedings of ICLS* (Vol. 98, pp. 35–41).

[CR38] Puntambekar S, Kolodner JL (2005). Toward implementing distributed scaffolding: Helping students learn science from design. Journal of Research in Science Teaching.

[CR39] Puntambekar S, Stylianou A, Goldstein J (2007). Comparing enactments of an inquiry curriculum: Lessons learned from two teachers. The Journal of the Learning Sciences.

[CR40] Quintana C, Reiser BJ, Davis EA, Krajcik J, Fretz E, Duncan RG (2004). A scaffolding design framework for software to support science inquiry. Journal of the Learning Sciences.

[CR41] Raes A, Schellens T, De Wever B, Vanderhoven E (2012). Scaffolding information problem solving in web-based collaborative inquiry learning. Computers & Education.

[CR42] Reiser BJ (2004). Scaffolding complex learning: The mechanisms of structuring and problematizing student work. The Journal of the Learning Sciences.

[CR43] Reiser BJ, Tabak I, Sawyer RK (2014). Scaffolding. The Cambridge handbook of the learning sciences.

[CR44] Reiser BJ, Tabak I, Sandoval WA, Smith BK, Steinmuller F, Leone AJ, Carver SM, Klahr D (2001). BGuILE: Strategic and conceptual scaffolds for scientific inquiry in biology classrooms. Cognition and instruction: Twenty-five years of progress.

[CR45] Remillard JT (2005). Examining key concepts in research on teachers’ use of mathematics curricula. Review of Educational Research.

[CR46] Saye JW, Brush T (2002). Scaffolding critical reasoning about history and social issues in multimedia-supported learning environments. Educational Technology Research and Development.

[CR47] Schofield JW, Eurich-Fulcer R, Britt CL (1994). Teachers, computer tutors, and teaching: The artificially intelligent tutor as an agent for classroom change. American Educational Research Journal.

[CR48] Smagorinsky P, Clayton CM, Johnson LL (2015). Distributed scaffolding in a service-learning course. Theory Into Practice.

[CR49] Smit J, van Eerde HAA, Bakker A (2012). A conceptualization of whole-class scaffolding. British Educational Research Journal.

[CR50] Songer NB, Fick S, Shah AM (2012). Characterizing teachers’ verbal scaffolds to guide elementary students’ creation of scientific explanations. School Science and Mathematics.

[CR51] Stemler S (2001). An overview of content analysis. Practical Assessment, Research & Evaluation.

[CR52] Tabak I (2004). Synergy: A complement to emerging patterns of distributed scaffolding. Journal of the Learning Sciences.

[CR53] Tabak I, Baumgartner E (2004). The teacher as partner: Exploring participant structures, symmetry, and identity work in scaffolding. Cognition and Instruction.

[CR54] Tabak, I., & Reiser, B. J. (1997). Complementary roles in software-based scaffolding and teacher-student interactions in inquiry learning. In R. Hall, N. Miyake, & N. Enyedy (Eds.) *Proceedings of Computer Support for Collaborative Learning’97* (pp. 289–298). Toronto, Ontario, Canada.

[CR55] Taub M, Azevedo R, Bouchet F, Khosravifar B (2014). Can the use of cognitive and metacognitive self-regulated learning strategies be predicted by learners’ levels of prior knowledge in hypermedia-learning environments?. Computers in Human Behavior.

[CR56] van de Pol J, Volman M, Beishuizen J (2010). Scaffolding in teacher-student interaction: A decade of research. Educational Psychology Review.

[CR57] van de Pol J, Volman M, Oort F, Beishuizen J (2014). Teacher scaffolding in small-group work: An intervention study. Journal of the Learning Sciences.

[CR58] Vygotsky, L. S. (1980). In M. Cole, V. John-Steiner, S. Scribner & E. Souberman (Eds.), *Mind in society: The development of higher psychological processes*. Cambridge, MA: Harvard University Press.

[CR59] Wood D, Bruner JS, Ross G (1976). The role of tutoring in problem solving. Journal of Child Psychology and Psychiatry and Allied Disciplines.

[CR60] Xenofontos N, Zacharia Z, Hovardas T (2018). How much guidance students need when designing experiments in a computer-supported inquiry learning environment. International Journal of Learning and Teaching.

